# Metagenomic characterization of microbial communities associated with alphaherpesvirinae detection in magnificent frigatebirds from French Guiana

**DOI:** 10.3389/fvets.2026.1866497

**Published:** 2026-10-14

**Authors:** Ilaria Di Bartolo, Manrico Sebastiano, Gabriele Vaccari, Elisabetta Suffredini, Arianna Boni, David Costantini, Luca De Sabato

**Affiliations:** 1 Department of Food Safety, Nutrition and Veterinary Public Health, Istituto Superiore di Sanità, Rome, Italy; 2 Behavioural Ecology and Ecophysiology Group, Department of Biology, University of Antwerp, Universiteitsplein, Wilrijk, Belgium; 3 Department of Ecological and Biological Sciences, University of Tuscia, Viterbo, Italy

**Keywords:** *Fregata magnificens*, frigatebird, herpesvirus, metagenomics, viral communities

## Abstract

**Introduction:**

A population of Magnificent Frigatebirds (Fregata magnificens, Suliformes) inhabiting French Guiana has been under investigation since 2005 due to high chick mortality rates associated with nodular proliferative skin lesions, characterized by featherless areas on the body. An initial study conducted in 2009 reported the presence of an alphaherpesvirus detected in a crust from a single individual in the same frigatebird population. However, the cause of the outbreaks and the associated high mortality in the frigatebird population remained undetermined.

**Methods:**

Almost 15 years later, in 2024, we collected beak swabs, cloacal swabs, and blood samples from 14 animals. Six animals were clinically healthy, while eight showed clinical signs including skin crusts, hyperkeratosis on the eyes, corneal thickening, and signs of distress. To investigate the potential causes of those signs, we screened the samples for herpesviruses. To characterize bacterial and viral communities and investigate potential co-infections, we selected four beak swabs for metagenomic analysis: two from herpesvirus-positive individuals and two from herpesvirus-negative controls (one healthy and one with clinical signs), and sequenced them using metagenomic Next Generation Sequencing (NGS).

**Results and discussion:**

Two individuals tested positive for herpesviruses in beak swabs, one healthy and one with clinical signs. Analysis of the bacterial community revealed that the most abundant phyla were Actinobacteria, Proteobacteria, Firmicutes, Bacteroidetes, and Fusobacteria, which typically inhabit the gut microbiota. All reported bacterial phyla were detected in every sequenced sample. At the viral level, among non-bacteriophage viruses, Orthoherpesviridae showed the highest abundance in PCR-positive animals, followed by Adenoviridae- and Hepadnaviridae-related sequences. Sequence analysis of the DNA-directed DNA polymerase (DdDp) region of Orthoherpesviridae identified two sequences: one with 98.1% nucleotide identity to sequences reported in the same geographic area in 2005, and the other with 72.1% identity to a vulture herpesvirus from India. The bacterial and viral populations detected were largely similar between animals with and without clinical signs. Due to the limited sample size, it was not possible to determine whether the detected microorganisms were associated with the observed clinical signs.

## 1 Introduction

Since 2005, a population of Magnificent Frigatebirds (*Fregata magnificens*, Suliformes) inhabiting Grand Connétable Island, a Nature Reserve in French Guiana, has been monitored due to the occurrence of numerous chicks exhibiting cutaneous clinical signs and increased mortality. An initial study conducted in 2009 identified a novel alphaherpesvirus strain in a skin crust sample from a single individual (1).

Herpesviruses are members of the family *Herpesviridae*. They are double-stranded DNA viruses currently classified into three subfamilies: *Alphaherpesvirinae*, *Betaherpesvirinae*, and *Gammaherpesvirinae*, according to updated ICTV classifications (2). A defining feature of herpesviruses is their ability to establish lifelong latent infections in their natural hosts, with viral reactivation commonly associated with immunosuppression or physiological stress (3). Certain herpesviruses can induce severe disease and significant mortality, particularly when transmission occurs across species barriers. In avian hosts, reported herpesvirus infections continue to be predominantly associated with Alphaherpesviruses, as confirmed by recent molecular and phylogenetic investigations (4).

Several studies have suggested that immunosuppression and environmental stressors (i.e., contaminant exposure and reduced food availability) may play a major role in disease susceptibility within frigatebird populations (5–7). Moreover, previous work found that chicks with severe visible clinical signs of the disease have high levels of inflammatory and oxidative damage markers (5, 8, 9) and modified expression of genes linked to immunity, metabolism and cell proliferation (10). However, these studies did not demonstrate a causal link between the detected virus and the disease outbreaks. Moreover, the virus was detected in oral swabs and skin lesions; however, these detections alone do not establish whether infection represented primary infection, latency, or reactivation.

Nearly 15 years after the first report of herpesvirus in frigatebirds from French Guiana (1), we re-examined the same population, prompted by the reappearance of similar lesions, for the presence of known or novel herpesvirus strains.

We examined the sampled animals using a pathogen-specific approach to detect the presence of herpesviruses, alongside a metagenomic approach to identify additional pathogens and characterize the viral and bacterial populations present in the frigatebirds investigated.

Over recent decades, the development of Next Generation Sequencing (NGS), particularly metagenomic approaches has enabled large-scale sequencing of microbial genomes and has greatly improved our understanding of bacterial and viral communities in humans and animals. However, relatively little is known about the bacterial and viral communities of wild bird species that lack economic importance. To date, no metagenomic studies have been conducted on frigatebird samples.

A comprehensive understanding of avian microbiota is therefore essential not only for identifying emerging infectious threats but also for elucidating the broader ecological and evolutionary roles of bacteria and viruses in wildlife systems.

In this study, we present an investigation of the oral microbiota of frigatebirds using metagenomic sequencing. We compared microbial communities between apparently healthy and affected individuals showing signs of disease with the aim of expanding current knowledge of microbial diversity in this species and its potential association with health status.

## 2 Materials and methods

### 2.1 Collection of samples

We carried out field work in January 2024 on Grand Connétable Island, a protected area off the Northern Atlantic coast of South America (French Guiana, 4°49’ 30” N; 51°56’ 00” W). We captured 14 chicks (1.5 to 3 months old) at the nest by hand. Six chicks were asymptomatic, did not show any visible clinical sign, and were thus considered healthy. Eight chicks showed signs of distress (e.g., lethargy) and severe and clear visible clinical signs of the disease which included skin crusts on the wings, neck, and head, hyperkeratosis on eyes and the consequent thickening of the cornea as described in de Thoisy et al. (1) and Sebastiano et al. (11). The severity of clinical signs was not scored as we selected birds with visible clinical signs of the disease, which was sufficient for the scope of the study. The work was carried out under permit EU-MNHN-UAE-FSNTC, n. A750536 and authorized by the Direction Générale des Territoire et de la Mer and Préfet De La Guyane, permit number R03-2023-11-24-00006. All birds were captured early in the morning or late in the afternoon to minimize stress and were released within a few minutes at their nests. We collected about 1 mL of blood from chicks using a syringe with a 25G needle from the brachial vein. Sera were left at 4 °C for 8 h and then separated by centrifugation at 3,000 × g for 5 min. We collected cloacal and beak swabs from all individuals. Furthermore, we opportunistically collected body crusts, and a broken piece of beak that fell from a very sick bird covered by crusts. Samples were immediately placed into empty sterile tubes without any transport or storage medium and kept in dry ice while on the field, and were then kept at −80 °C until laboratory analyses.

### 2.2 Herpesvirus detection

We extracted total DNA using the Nuclisens Mini Mag (Biomerieux, Bagno a Ripoli, FI, Italy) and either stored it at −80 °C or used it immediately. To screen for herpesvirus presence, we performed a broad-range nested PCR following the thermal profile designed by VanDevanter et al. (12), using GoTaq G2 Flexi DNA Polymerase (Promega, Milan, Italy), following the manufacturer’s instructions, to amplify a short genomic fragment (approximately 215 to 315 bp) within the DNA-directed DNA polymerase (DdDp) gene. We analyzed the PCR products on a 2% agarose gel. Eurofins Genomics (Ebersberg, Germany) sequenced the DNA amplicons using the original PCR primers.

The two herpesvirus-positive beak swab samples identified through broad-range PCR and subsequent NGS (Section 2.3) were characterized further using two additional sequence-specific PCR assays designed based on the NGS-derived DdDp sequences. These sequence-specific assays targeted the herpesvirus sequences identified in samples 1C and 9C, respectively. These assays were applied to all samples, also those negative for the broad-range PCR, to confirm the sequence-level distribution and to verify the absence of these specific sequences in PCR-negative individuals.

To this purpose we used the online tool Primer3web to design the following primer pairs to amplify a short genomic region within the DdDp gene of approximately 596 bp, which BioFab Research (Rome, Italy) synthesized: 1C assay: Fw 5’-GGAGATAACGGTAGGGGACG-3′, Rw 5’-ATCATCTTCCCGCCGTGTAT-3′, 9C assay: Fw 5’-TTTCGTCAGACCGCACATAC-3′, Rw 5’-CCCACCATGTATCACGCCTA-3′.

PCR amplifications were performed using GoTaq G2 Flexi DNA Polymerase (Promega) following the manufacturer’s instructions. The thermal cycling profile consisted of initial denaturation at 95 °C for 2 min; 40 cycles of denaturation at 95 °C for 30 s, annealing at 55 °C for 30 s, and extension at 72 °C for 1 min; followed by a final extension at 72 °C for 5 min.

### 2.3 Statistical analyses

Herpesvirus prevalence and corresponding 95% confidence intervals (95% CI) were calculated using the exact binomial method, which is appropriate for small sample sizes and rare events (*n* < 30). Animals were classified as herpesvirus-positive if herpesvirus was detected in at least one sample type (serum, crust, beak swab, or cloacal swab). Prevalence estimates were stratified by health status (healthy vs. sick). Differences in herpesvirus prevalence between groups were compared using Fisher’s exact test with α = 0.05 as the significance threshold. All statistical analyses were performed in R Studio v.2026.06.0 using the binom package.

### 2.4 Metagenomic sequencing

Following broad-range nested PCR screening for herpesvirus, we selected four beak swab samples for metagenomic sequencing. The selection included: two herpesvirus-positive samples (one from a healthy individual [1C] and one from a diseased individual [9C]) and two herpesvirus-negative samples randomly chosen (one from a healthy individual [5C] and one from a diseased individual [6C]).

We quantified the extracted DNA using the Qubit 2.0 Fluorometer (Thermo Fisher Scientific) with the Qubit dsDNA high sensitivity (HS) kit. We prepared libraries using the NEBNext Fast DNA Library Prep Set for Ion Torrent (New England BioLabs, Milan, Italy), for the enzymatic fragmentation, and we performed size selection for fragments of approximately 400 bp, matching the specific limits of the 400-base chemistry kit of Ion Torrent machines and following the manufacturer’s instructions. We performed the emulsion PCR and the sequencing run using the Ion 520 & Ion 530 Kit-OT2 on the Ion Gene Studio S5 (Thermo Fisher Scientific, Milan, Italy), according to the manufacturer’s instructions. We ran two samples in pairs on an Ion 530 Chip to obtain approximately 10 million reads per sample.

### 2.5 Bioinformatic analysis

Raw sequencing reads generated by the Ion Gene Studio S5 were assessed for quality metrics using FastQC v0.11.9 (13). Reads were trimmed using Trimmomatic v0.39 (14) with the following parameters: LEADING:3, TRAILING:3, SLIDINGWINDOW:4:15, and MINLEN:50, retaining reads ≥50 bp. *De novo* metagenomic assembly was performed using metaSPAdes v3.15.5 (15) with k-mer sizes of 21, 33, 55, 77, 99, and 127 bp. The --iontorrent flag was specified to optimize assembly parameters for Ion Torrent sequencing, which is particularly important for correcting homopolymer-associated indels characteristic of this platform. Assembly was conducted on a local desktop with 64 GB RAM and 8 processors. Contigs shorter than 500 bp were excluded to remove assembly artifacts and low-coverage sequences.

Metagenomic contigs were taxonomically classified using DIAMOND v0.8.24 (16) (*e*-value threshold <0.001) against a comprehensive reference database. The database was constructed by concatenating protein sequences from three reference sources downloaded from NCBI-nr (Non-redundant protein database) via Entrez search, filtered by the following taxonomy identifiers: *Aves* (taxonomy ID 8782), Bacteria (taxonomy ID 2), and Viruses (taxonomy ID 10239). The final database (created in January 2026) included 6,367,021 protein sequences for *Aves*, 721,995 for Viruses and 401,294,419 for Bacteria. Metagenomic contigs were assigned to taxonomic categories using MEGAN version 6 (17) with default parameters. DIAMOND protein sequence alignments (from previous analysis) were used as input to MEGAN, and taxonomic assignments were established using the prot-gi2taxid mapping file to convert NCBI protein identifiers to NCBI taxonomy identifiers.

Contigs with significant sequence similarity (*e*-value <0.001) to *Aves* sequences were classified as host-derived and subsequently excluded from metagenomic and ecological analyses. Bacterial and viral contigs were retained for downstream community profiling.

Taxonomic classification results were expressed as percentages of total contigs for kingdom-level assignments (Bacteria, Viruses). For lower taxonomic ranks (phylum, class, family, genus), percentages were calculated relative to the total number of contigs assigned to the corresponding parent taxon. Only taxonomic assignments representing ≥1% of their respective higher-level category were included in reported results, to focus analyses on dominant members of the microbial and viral communities.

Because sequencing of negative (reagent-only) controls was not performed, potential reagent-derived contaminant sequences could not be formally identified. Six low-abundance genera reported as common reagent/kit contaminants (*Burkholderia*, *Bradyrhizobium*, *Pseudomonas*, *Cutibacterium*, *Achromobacter*, and *Rhizobium*) were therefore retained in all downstream alpha- and beta-diversity analyses, as no data-driven basis was available to justify their exclusion. This decision was not based on relative abundance as a decontamination criterion, low abundance alone does not distinguish contamination from genuine rare taxa, but reflects the absence of a validated method to make that distinction in this dataset.

### 2.6 Alpha and Beta diversity analysis

Alpha diversity metrics were calculated at the genus taxonomic level for bacterial communities to characterize community richness and evenness. Three complementary indices were computed:

1. Shannon diversity index (H). This index incorporates both richness (number of taxa) and evenness (relative abundance distribution).
2. Simpson’s dominance index (D), calculated as *D* = Σpi^2^ (where pi is the relative abundance of genus i), which quantifies the probability that two randomly selected contigs belong to the same genus. Higher D values indicate greater dominance by one or a few genera (lower evenness); lower values indicate that abundance is more evenly distributed among genera, with no single genus strongly dominating the community.
3. Richness, determined as the count of distinct bacterial genera with non-zero abundance in each sample.

Relative abundance for diversity calculations was defined as the percentage, per sample, of bacterial contigs classified to the genus level (after exclusion of host-derived and viral sequences), normalized to sum to 100% across all genera detected in that sample. Bacterial contigs assigned to Bacteria but not resolved to genus level (i.e., classified only at a higher taxonomic rank) were not included in the genus-level relative-abundance distribution and were therefore not used in the calculation of Shannon and Simpson’s indices.

Beta diversity analysis evaluated compositional differences between samples using:

1. Bray-Curtis distance, calculated as BC = Σ|xi - yi| / Σ(xi + yi), where xi and yi represent the relative abundances of genus i in samples x and y, respectively. This metric ranges from 0 (identical communities) to 1 (completely different communities).
2. Principal Coordinate Analysis (PCoA) was performed on the Bray-Curtis distance matrix using eigenvalue decomposition to visualize sample positioning in a reduced-dimensional ordination space and to identify clustering patterns or compositional gradients among samples.

Viral communities were not subjected to formal alpha and beta diversity analysis due to limited genus-level representation (*n* = 3 genera detected), which falls below the recommended minimum threshold (≥10 taxa) for statistically meaningful diversity quantification.

### 2.7 BLAST searches and phylogenetic analysis

After the taxonomic assignment of contigs performed using DIAMOND against the NCBI non-redundant protein database, followed by taxonomic binning with MEGAN, those assigned to taxa of specific interest were further inspected using BLAST and phylogenetic analysis for confirmation.

The phylogenetic analyses of the Adenoviridae- and Hepadnaviridae-related sequences did not yield either sufficiently reliable alignments or statistically supported topologies to allow taxonomic inference. The reasons were linked to short contig lengths obtained, the absence of informative conserved regions and the genomic regions recovered which displayed a limited similarity to available reference sequences.

For phylogenetic analysis, due to the high nucleotide differences between sequences obtained in this study and herpesvirus sequences on NCBI database, we built a dataset using protein sequences of DdDp Alphaherpesviruses. The dataset included: 2 sequences from this study, the 2 closest sequences from NCBI database, 52 Alphaherpesvirus sequences from RefSeq database (non-redundant), 1 Betaherpesvirus sequence as outgroup (NP_042931.1) (Supplementary Table 1).

We performed sequence alignment using mafft v.7.526 (11), run locally using the “--auto” option, to select the appropriate alignment strategy, and more accurate strategies such as L-INS-i and E-INS-i, and the alignment was manually inspected and refined to ensure optimal alignment quality. The alignment was visualized using Aliview v.1.30 (18) for a total length of 268 amino acids. However, despite the different strategies, phylogenetic inference was not possible for some sequences. For the phylogenetic analysis, we constructed a maximum likelihood tree using IQ-TREE 2 (19), employing the model suggested by ModelTest (Q.insect+F + I + G4: Quartet insect, stationary amino-acid frequencies, invariant sites and Gamma distribution) with 1,000 bootstrap replications.

## 3 Results

### 3.1 Herpesvirus screening of biological samples from frigate birds

Herpesvirus was detected in 2 of the 14 animals examined (14.3%; 95% CI: 1.8–42.8%) (Table 1). Both positive animals were identified via beak swabs. When stratified by health status, herpesvirus prevalence was 16.7% (1/6; 95% CI: 0.4–64.1%) in healthy animals and 12.5% (1/8; 95% CI: 0.3–52.7%) in sick animals. No significant difference in herpesvirus prevalence was observed between the two groups (Fisher’s exact test, *p* = 1.00) (Table 2). Herpesvirus detection varied considerably by sample type. Beak swabs showed a prevalence of 14.3% (2/14) and 20.0% in crust samples (1/5). However, crust samples had a higher rate of missing data (9/14 animals). Serum samples were uniformly negative (0/12), and no herpesvirus was detected in cloacal swabs (0/14). The healthy herpesvirus-positive animal (1C) tested positive only in the beak swab, while the positive sick animal (9C) tested positive in both beak and crust samples.

**Table 1:** Summary of epidemiological data available for each animal and results obtained by pan Herpesvirus-PCR.

ID animal	Status	Age (months)	Serum	Crust	Beak swab	Cloacal swab
1C	Healthy	2.5	0	*	1	0
2C	Healthy	1.5	0	*	0	0
3C	Healthy	1.5	0	*	0	0
4C	Healthy	3	0	*	0	0
5C	Healthy	3	0	*	0	0
6C	Sick	2.5	0	0	0	0
7C	Sick	3	0	0	0	0
8C	Sick	2	0	0	0	0
9C	Sick	2	0	1	1	0
10C	Sick	3.5	0	0	0	0
11C	Sick	2.5	0	*	0	0
12C	Healthy	2	0	*	0	0
13C	Sick	3	*	*	0	0
14C	Sick	1.5	*	*	0	0

*Specimen not available; 1 positive; 0 negative.

**Table 2:** Herpesvirus prevalence and detection by health status.

Group	Animals (#)	Positive (#)	Prevalence	95% CI
Healthy	6	1	16.7%	0.4–64.1
Sick	8	1	12.5%	0.3–52.7
Serum	12ᵃ	0	0%	0.0–26.5
Crust	5^b^	1	20.0%	0.5–71.6
Beak swab	14	2	14.3%	1.8–42.8
Cloacal swab	14	0	0%	0.0–24.2
Total	14	2	14.3%	1.8–42.8

ᵃTwo serum samples were not available. ^b^Nine crust specimens were not available. CI, confidence interval; exact binomial method used.

The sequences obtained from the beak swab and crust of animal 9C were identical. The BLASTn searches reported the highest nucleotide identity (78.7% nt.id.) with *Aquila chrysaetos* herpesvirus strain 367/19 (MW533128.1) reported in Slovenia in 2019 in the golden eagle (*Aquila chrysaetos*). The sequence from 1C showed the highest identity with *Fregata magnificens* herpesvirus DNA polymerase gene, partial cds (98.3% nt.id., EU867220.1) reported in a study conducted in French Guiana in 2005. Both sequences were related to Herpesvirales sequences classified within the subfamily Alphaherpesvirinae.

The sequence from 1C showed 64.6% nucleotide identity and 63.3% amino acid identity with the sequence from 9C.

### 3.2 Metagenomic analysis

To characterize the bacterial and viral populations within the frigatebirds, we selected and analyzed beak swab samples from four individuals. Two animals tested positive for herpesviruses by PCR: one healthy chick (1C) and one sick chick presenting crusts (9C). The remaining two samples randomly chosen, one from a healthy chick (5C) and one from a sick chick (6C), tested negative for herpesviruses via PCR.

Information on the number of reads and contigs produced in this study, before and after quality checking, is reported in Table 3.

**Table 3:** Sequencing and assembly statistics.

Sample	1C	5C	6C	9C
Sequencing reads				
Raw reads (#)	13,976,812	10,399,718	11,314,472	8,783,822
Raw reads (length range, bp)	25–613	25–603	25–583	25–616
Quality-filtered reads (#)	12,710,330	8,783,939	10,426,395	8,427,304
Quality-filtered (length, bp)	50–537	50–603	50–583	50–616
Retention rate (%)	91.0	84.5	92.1	95.9
Assembly (Pre-contig filtering)				
Contigs (#)	205,242	181,442	815,770	381,470
Total length (bp)	156,463,183	96,722,082	550,437,561	262,329,689
N50	1,518	2,714	1,282	1,169
L50	15,651	4,303	102,608	47,993
Assembly (Post-filtering <500 bp)				
Contigs (#)	123,725	71,824	480,733	240,378
Total length (bp)	136,635,308	69,889,602	469,081,655	228,134,620
N50	1,518	2,714	1,282	1,169
L50	15,651	4,303	102,608	47,993
Contigs removed (%)	60.3	60.4	41.1	37.0

We classified contigs as belonging to birds, to exclude host sequences and then further classified them as bacterial or viral as reported in Table 4.

**Table 4:** Summary of taxonomic classification of contigs from beak samples.

Sample ID	Total contigs	Birds *n* (%)	Bacteria *n* (%)	Viral *n* (%)
1C	123,725	50,167 (40,5)	40,966 (33,1)	1,022 (0,8)
5C	71,824	20,043 (27,9)	29,963 (41,7)	505 (0,7)
6C	480,733	332,523 (69,2)	29,705 (6,2)	636 (0,1)
9C	240,378	143,306 (59,6)	34,105 (14,2)	1,007 (0,4)

### 3.3 Bacterial taxonomic profiling

The number of contigs classified as bacterial was: 40,966 for 1C, 29,963 for 5C, 29,705 for 6C, and 34,105 for 9C (Table 4).

In the metagenomes assayed, 25 phyla were identified (Supplementary Figure 1). The most common were Actinobacteria (39.0–54.0%), Proteobacteria (4.9–16.0%), Firmicutes (27.0–31.0%), Bacteroidetes (7.3–12.0%), Fusobacteria (1.4–6.1%), all of which were detected in every sample. The remaining phyla each accounted for less than 0.3%.

At the class level, a total of 53 different classes were detected, 28 of which were found in all 4 samples. Twenty-five of them were missing in at least one sample (Supplementary Figure 2).

Among the phylum Actinobacteria, the most abundant class was the Actinomycetia (43–59%). Within the Firmicutes the most abundant was Negativicutes (13.0–19.0%), within Bacteroidetes the Bacteroidia (4.6–10.0%), and within Proteobacteria the Gammaproteobacteria (1.3–11.0%). The remaining classes each accounted for less than 0.6%.

The most abundant classes were further examined at the family level. Figure 1 reports families for each class with a relative abundance above 1%.

**Figure 1:**
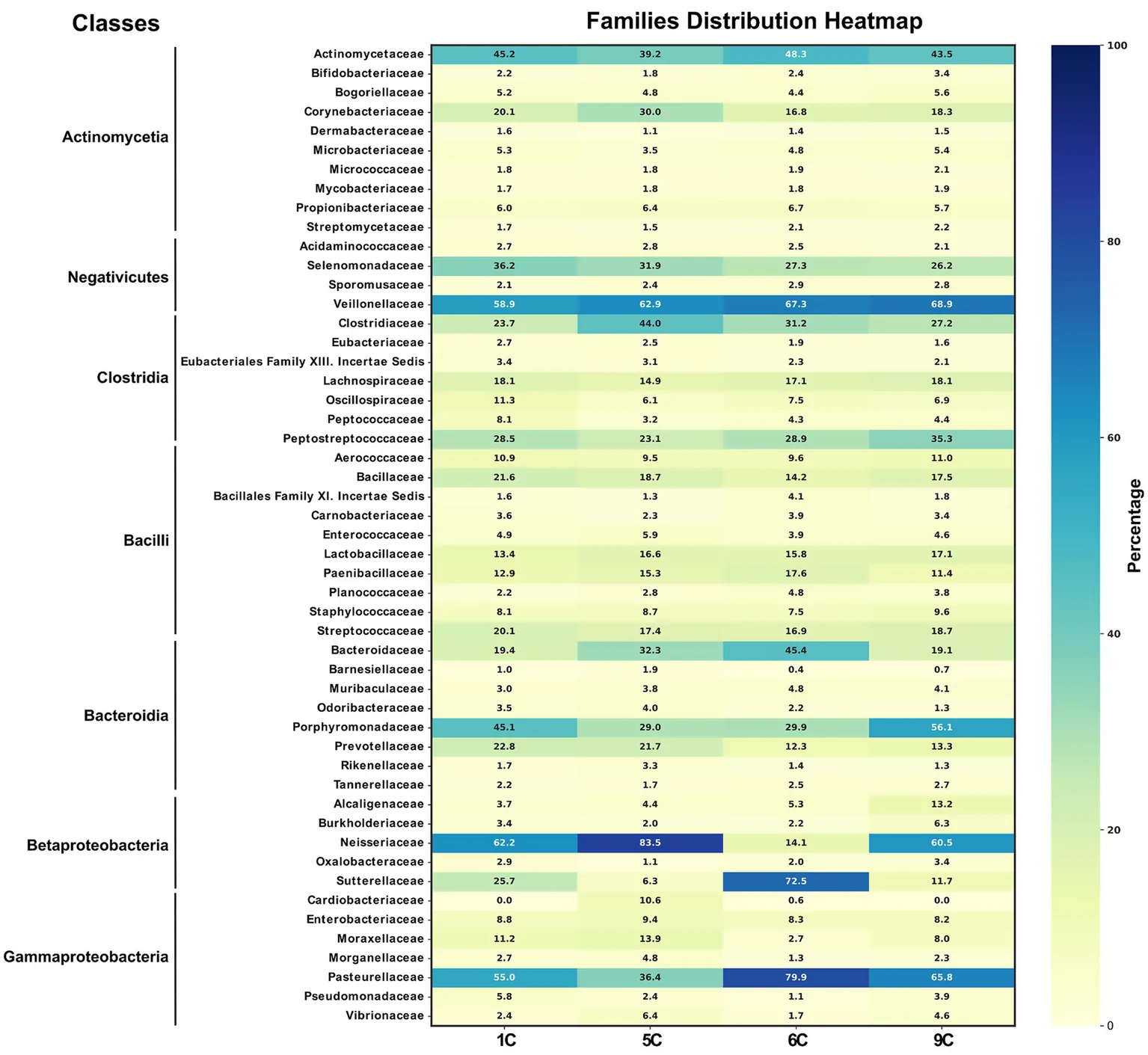
Heatmap illustrating the relative abundance (percentage) of contigs classified at the family level for each individual.

The families with the highest percentage were: among the Actinomycetia class Actinomycetaceae (39.2–48.3%), among the Negativicutes class the Veillonellaceae (58.9–68.9%), among the Clostridia class, Clostridiaceae (23.7–44.0%), among the Bacilli class, there were Bacillaceae (14.0–22%), among the Bacteroidia class, the most abundant families were Porphyromonadaceae (29.0–56.1%), among Gammaproteobacteria: Pasteurellaceae (55.0–80.0%), and for Betaproteobacteria: Neisseriaceae (14.0–62.0%) and Sutterellaceae (6.3–72.0%).

The most abundant classes and families were present in both healthy and sick animals.

#### 3.3.1 Bacterial community diversity

To assess bacterial community structure and diversity differences among samples, alpha and beta diversity indices were calculated at the genus level, revealing 546 genera in total.

##### 3.3.1.1 Alpha diversity

Alpha diversity analysis revealed broadly similar levels of bacterial genus-level diversity across the four samples (Supplementary Table 2). The Shannon diversity index, which measures both richness and evenness of the community, ranged narrowly from 4.02 (sample 6C) to 4.10 (sample 1C). Sample 1C exhibited the highest Shannon index (H = 4.10), indicating the most even distribution of bacterial genera, while sample 6C showed the lowest Shannon diversity (H = 4.02). Samples 5C and 9C displayed intermediate, nearly identical Shannon values (H = 4.09 for both), suggesting similar diversity levels.

Genus richness (the number of distinct genera detected) varied substantially among samples, with sample 5C displaying the highest richness (363 genera), followed by sample 6C (324 genera) and sample 9C (314 genera), while sample 1C showed the lowest (285 genera). Simpson’s dominance index was low across all samples (*D* = 0.047–0.059), indicating that no single genus achieved strong numerical dominance in any sample. Sample 1C showed the lowest dominance (*D* = 0.047), while sample 5C showed the highest (*D* = 0.059), indicating that, despite its high richness, sample 5C had a comparatively less even distribution of abundance among genera.

##### 3.3.1.2 Beta diversity

Beta diversity analysis using Bray-Curtis distance revealed compositional differences between samples (Supplementary Table 3). The distance matrix showed moderate dissimilarity across pairwise sample comparisons, ranging from 0.151 to 0.292. Samples 1C and 9C displayed the lowest dissimilarity (BC = 0.151), indicating the most similar bacterial community composition. In contrast, samples 5C and 6C were the most dissimilar (BC = 0.292), suggesting the greatest compositional differences. Additional pairwise comparisons showed intermediate dissimilarities: 1C vs. 5C (BC = 0.209), 1C vs. 6C (BC = 0.284), 5C vs. 9C (BC = 0.215), and 6C vs. 9C (BC = 0.265).

The Principal Coordinate Analysis (PCoA) plot (Supplementary Figure 3) visualized the ordination of the four samples, with the first two principal coordinates explaining approximately 87.4% of the total variance. Sample 9C clustered most closely with sample 1C, consistent with their low Bray-Curtis distance, while sample 6C occupied a more distinct position in the ordination space. Sample 5C, despite having the highest genus richness, showed moderate dissimilarity to all other samples, suggesting a distinct genus composition.

### 3.4 Viral taxonomic profiling

The number of contigs classified within the viral kingdom was 1,022 in 1C, 505 in 5C, 636 in 6C, and 1,007 in 9C.

A total of 8 classes were detected, 4 of which were present in all samples: Caudoviricetes (52.6–95.0%), Malgrandaviricetes (2.0–4.8%) and Megaviricetes (1.3–42.8%), all identified as bacteriophages and therefore excluded from further analysis. The class Herviviricetes (26.0–34.4%) was observed only in samples 1C (healthy) and 9C (sick), represented solely by family *Orthoherpesviridae*. The remaining classes: Pharingeaviricetes, including only the family *Adenoviridae*, Revtraviricetes including only the family *Hepadnaviridae* in samples 1C and 9C, were present in at least one sample at a relative abundance below 1.0%. Diversity indices were not calculated for the viral communities, as the three non-bacteriophage viral families detected did not provide sufficient statistical power for meaningful alpha and beta diversity analysis.

#### 3.4.1 Orthoherpesviridae

For sample 1C, we assembled a contig of 134,121 nt, and for sample 9C, one of 966 nt in length, both classified within the family *Orthoherpesviridae*. The short DdDp sequences obtained via pan-herpesvirus PCR showed 100% nucleotide identity with the DdDp sequences found in these two contigs. Using the megaBLAST algorithm, the 1C sequence showed the highest identity (98.3%) to a 483 bp fragment of the *Fregata magnificens* herpesvirus DNA polymerase gene (EU867220.1). Using BLASTn, the best match, considering query coverage, was the Sphenicid alphaherpesvirus 1 isolate lib01004 genome (NC_033464.1), with 64.9% nucleotide identity, primarily within the DdDp region (Query coverage: 27.0%, *e*-value: 0, Alignment Length: 4144 nt).

ORF Finder identified 79 partial or complete open reading frames (ORFs). By comparing the proteins derived from these ORFs against the NCBI *Orthoherpesviridae* protein database, we confirmed that 78 ORFs belonged to this family, with amino acid distances ranging from 29.7 to 73.4% (Figure 2). Because we recovered a nearly complete genome, we did not observe the terminal repeats (TR) and internal repeats (IR) typical of the *Orthoherpesviridae* family at the 5′ and 3′ ends.

**Figure 2:**
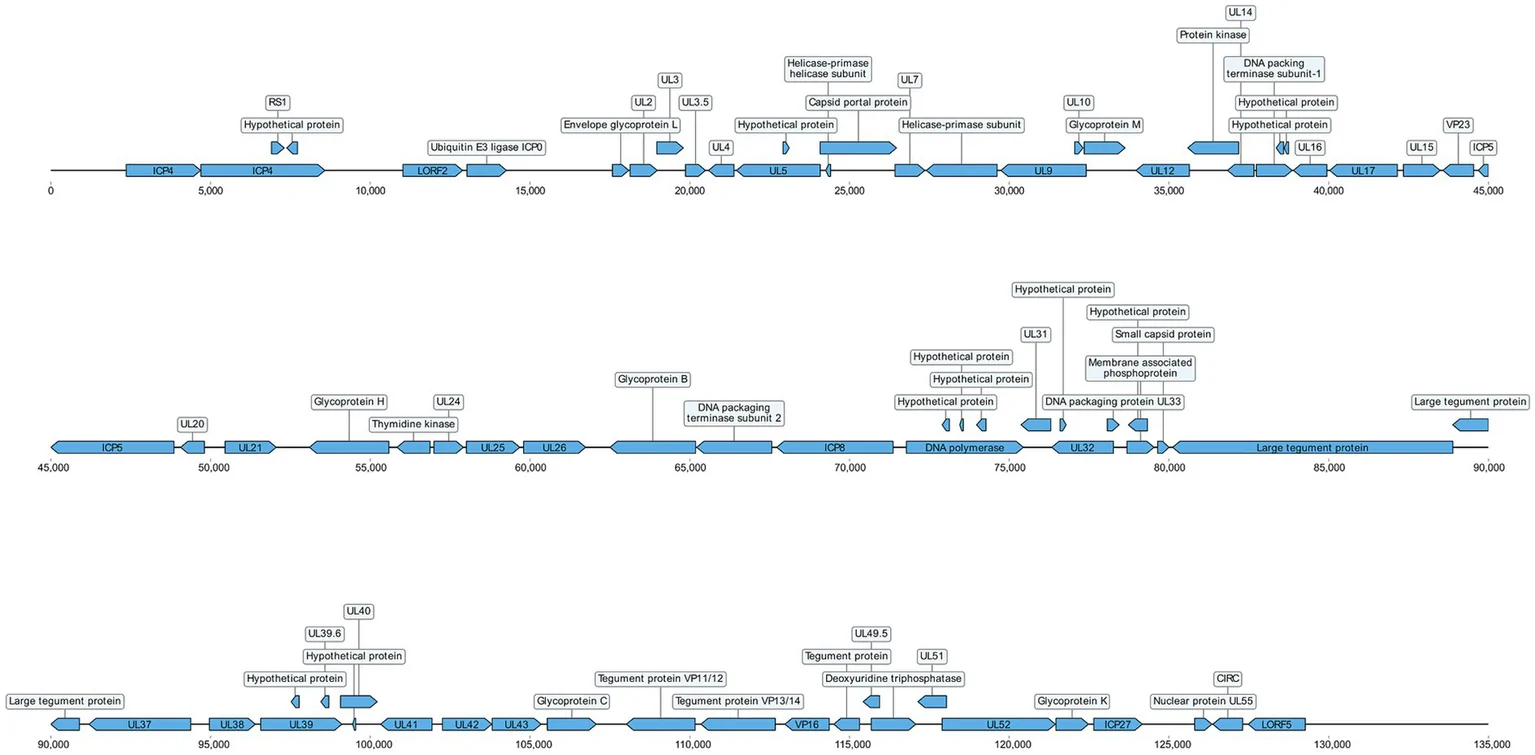
Schematic presentation of the 1C herpesvirus nearly complete genome organization.

For the 9C contig, using the BLASTn algorithm, the best match was the Vulture herpesvirus DNA polymerase (UL30) gene (AY571851.1), with 72.1% nt.id (Query coverage: 92.0%, *e*-value: 4e^−131^, Alignment Length: 891 nt).

The maximum likelihood phylogenetic tree (Figure 3), constructed using Alphaherpesvirales UL30 partial sequences, clustered the 1C and 9C sequences with two previously reported avian herpesviruses, the Vulture herpesvirus (AAT79466.1) reported in India and the Spheniscid alphaherpesvirus 1, identified in African penguins in 2020. Three additional trees based on helicase-primase subunit (UL5), capsid portal protein (UL6), and the complete UL30 protein sequence, excluding the partial 9C sequence, were built and showed the same topology as the first tree (Supplementary Figures 4–6).

**Figure 3:**
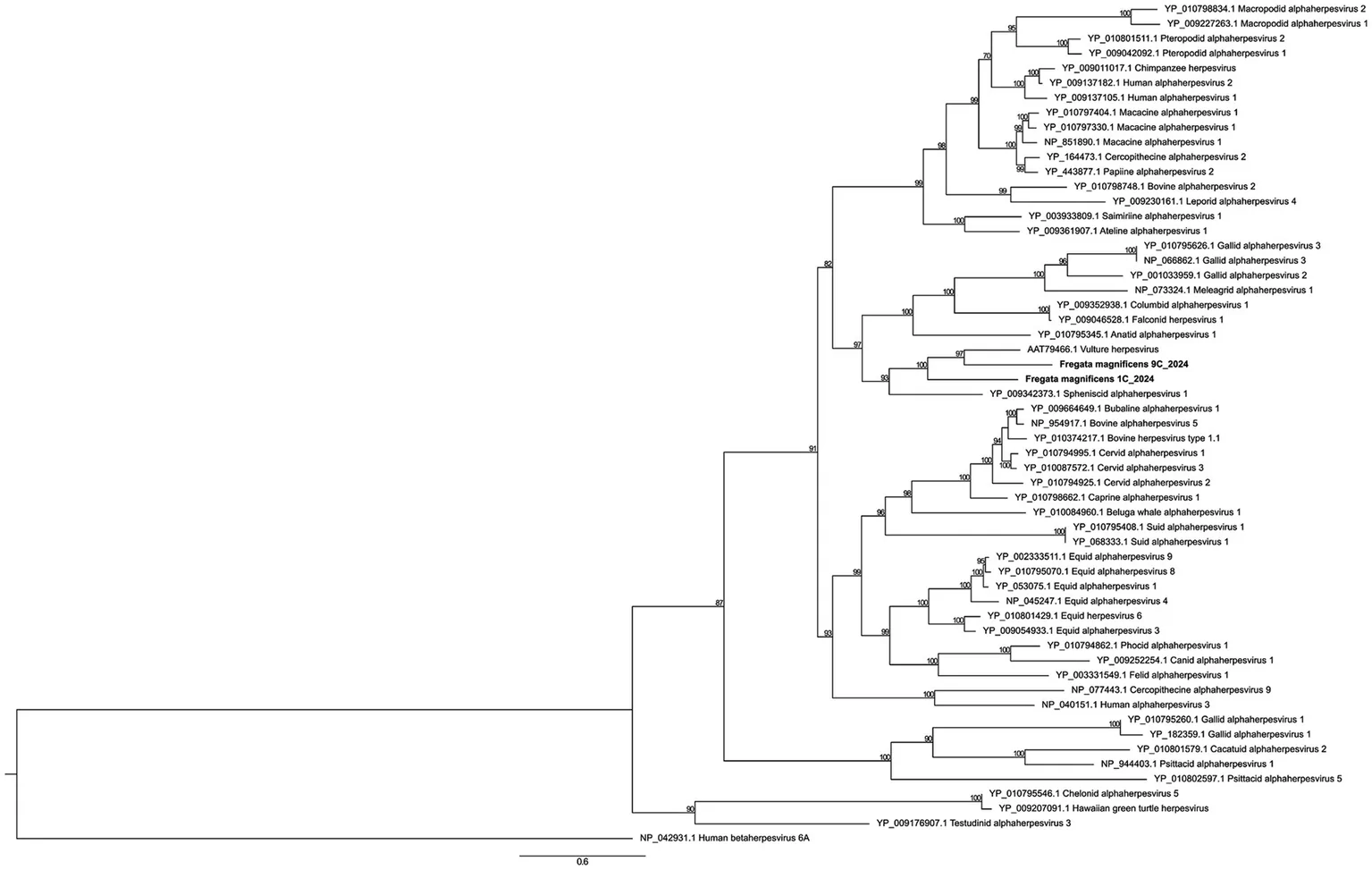
Phylogenetic Maximum Likelihood tree was built using an amino-acid alignment of partial DdDp sequence (UL30 genomic region) from Alphaherpesvirus. The analysis included the sequences obtained from samples 1C and 9C. Branch support values were calculated using 1,000 bootstrap replicates and model chosen by ModelTest (Q.insect+F + I + G4).

We re-screened all samples using the specific primers designed in this study, based on the metagenomic results, targeting either the 1C or 9C alphaherpesvirus sequences specifically.

These secondary assays were conducted to confirm the sequence-level distribution and to identify potential false-negative results that may have arisen from the use of less specific PCR primers in the initial screening. As expected, these secondary PCRs confirmed results for animal 1C, in the beak, and 9C in both the beak swab and the crust samples. Conversely, samples 5C and 6C tested negative, confirming the initial results obtained with the broad-range pan-herpes PCR. No other samples tested positive for herpesvirus.

Sequence analysis confirmed that the 9C beak and crust sequences were identical to each other and to the sequences obtained through NGS.

#### 3.4.2 Virus-related sequences

The similarities with virus-related sequences were obtained by BLASTx similarities search.

We identified one contig from sample 1C (550 bp) and one from sample 9C (926 bp) considered *Adenoviridae*-related sequences showing amino-acid similarity to *Aviadenovirus* ORF19 proteins.

The contig from 1C matched the ORF19 protein of a strain previously reported in a 2020 metagenomic study of wild *Psittacara leucophthalmus* in Brazil (NC_076721.1) with 36.2% amino acid identity (Query coverage: 99.0%, *e*-value 5e^−23^, Alignment Length: 185 aa).

The contig from 9C aligned with the ORF19 of Pigeon adenovirus 2, reported in *Columba livia* in Poland in 2023 (PP999623.2) with 44.0% amino acid identity (Query coverage: 94.0%, *e*-value 7e^−49^, Alignment Length: 232 aa).

In addition, we identified one contig in sample 1C (571 bp) and one in sample 6C (706 bp), considered *Hepadnaviridae*-related sequences showing amino-acid similarity to *Hepadnaviridae* polymerase proteins.

The contig from 1C matched the polymerase protein of a hepatitis B virus strain isolated from a female Mandarin duck in the USA (AAR89924), with 59.7% amino acid identity (Query coverage: 59.0%, *e*-value 1e^−09^, Alignment Length: 62 aa). In sample 6C, one contig matched a polymerase protein sequence from an Italian *Lonchura striata* (YBO75807) with 50.0% amino acid identity (Query coverage: 98.0%, *e*-value 1e^−69^, Alignment Length: 230 aa).

## 4 Discussion

Following the initial 2005 survey of herpesviruses in French Guianese frigatebirds (1), we investigated viral circulation within the same population in 2024. To identify potential causes of visible clinical symptoms and chick mortality, we characterized the broader bacterial and viral communities.

Using pan-herpesvirus and sequence-specific PCRs, we identified two herpesvirus-positive individuals: one healthy and one symptomatic. Sequencing revealed two distinct DdDp sequences. The first sequence, from individual 1C, shared high nucleotide identity (98.0%) with the sequence identified in this population in 2005 (1) and 2015 (5). The detection of two nearly identical sequences across almost two decades supports its endemic circulation. While previous studies associated this sequence with symptomatic animals, we detected it here in a healthy individual. This finding indicates that detection of this viral sequence is not restricted to symptomatic animals; however, PCR- and sequencing-based detection alone cannot determine whether the virus was actively replicating, latent, or undergoing reactivation at the time of sampling, and we therefore do not infer its replicative state in this individual. Furthermore, a similar alphaherpesvirus (97.0% nt.id.) circulates in *Fregata magnificens* in Brazil, without being associated with high mortality; together with its detection here in a healthy individual, this observation indicates that detection of this sequence alone cannot be interpreted as evidence of a causal role in disease occurrence in this population.

Our results showed the co-circulation of two distinct herpesvirus sequences in the population.

The DdDp sequence retrieved from a symptomatic individual (9C) was identified for the first time in this study and related to a herpesvirus sequence previously detected in *Aquila chrysaetos* (20) and showed 64.6% nucleotide identity to the DdDp sequence reported in this frigatebird population in 2005 (1). Although the absence of this sequence from previous surveys could be consistent with a more recent introduction into the population, the available data are insufficient to distinguish this possibility from previously undetected circulation.

Detection of herpesvirus DNA in beak swabs and skin crusts confirms the presence of viral DNA at these sampling sites but does not, by itself, demonstrate active viral replication or establish that herpesvirus caused the observed lesions. Conversely, a negative PCR result cannot completely exclude infection in either clinically affected or apparently healthy individuals. Viral latency, temporal variation in shedding, low viral load, sampling-site differences, and assay sensitivity may all contribute to negative results (20, 21). Therefore, the relationship between herpesvirus detection and the observed clinical signs should be interpreted as a possible association rather than evidence of causation.

The absence of the virus in sera could indicate an absence of viremia. Formulating a hypothesis for this is difficult since researchers have described herpesvirus in the blood in birds, only in cases of Pacheco’s disease, a highly infectious and acute disease caused by *Psittacid alphaherpesvirus 1* (PsHV-1) (22), and in non-avian species only for Canine Herpesvirus (CHV-1) in dogs (23) and Equine Herpesvirus (EHV-1) in horses (24). Moreover, due to the limited sample size it is difficult to link clearly the clinical signs to herpesvirus detection.

Few studies have reported the presence of herpesvirus in wild birds. For instance, a study from Slovenia (20) identified herpesviruses in 11 species from six different avian orders: Accipitriformes, Charadriiformes, Columbiformes, Falconiformes, Passeriformes, and Strigiformes. Our phylogenetic analysis shows that various herpesvirus sequences circulate within wild bird populations. While some herpesviruses are host species–specific, researchers have detected very similar sequences across different avian orders, indicating that herpesviruses are not always restricted to specific bird species (25).

From NGS data analysis, we obtained a nearly complete genome from the healthy animal (1C) and a short contig within the DdDp region (9C). The analysis of the longer sequences allowed a higher resolution phylogenetic analysis, establishing an evolutionary relationship with a Vulture herpesvirus reported in India and with a Spheniscid alphaherpesvirus, identified in African penguins in 2020. Few studies have reported circulation of *Alphaherpesvirus* in birds, including *Turdid alphaherpesvirus 1* in passerines (26), *Gallid alphaherpesvirus 1* (the causative agent of avian infectious laryngotracheitis in domestic fowl) (27), *Passerid herpesvirus 1* in Gouldian finches (*Chloebia gouldiae*) (28), and *Falconid herpesvirus 1* (FaHV-1), the etiological agent of inclusion body disease in falcons (29).

Because one sample from an apparently healthy chick and one from a visibly sick animal tested positive for herpesvirus, we used a metagenomic approach to sequence two additional animals, one healthy and one sick, both negative for herpesvirus, to investigate the presence of other pathogens and characterize the viral and bacterial communities.

A key limitation in interpreting the metagenomic results is that we relied heavily on reference data from intestinal microbiota studies to interpret oral microbial communities, due to the absence of metagenomics studies on the oral communities of frigatebirds. In addition, we drew comparisons to the most relevant available literature on avian microbiota, which is predominantly derived from fecal or gastrointestinal samples. While this approach provided a useful framework for interpreting our findings, we acknowledge that oral microbiota may exhibit distinct compositional and functional characteristics compared to intestinal microbiota. In addition, as sequencing of negative controls was not performed, low-abundance and known reagent-derived contaminants genera (*Burkholderia*, *Bradyrhizobium*, *Pseudomonas*, *Cutibacterium*, *Achromobacter*, and *Rhizobium*, all <0.01% relative abundance) (30) could not be conclusively distinguished from rare, genuine community members. These taxa were retained in all diversity and community analyses because no reagent-blank data were available to support their removal; their inclusion is therefore an unresolved limitation of this study rather than a validated methodological choice, and we do not draw any biological interpretation from their presence in our samples.

Among the bacterial populations, the phyla Proteobacteria, Firmicutes, Actinobacteria and Bacteroidetes that we observed in frigatebird samples were also the most abundant in wild passerines, and in Cooper’s hawks (31). In general, these phyla are the most common in fecal or gut samples, as previously described in 59 Neotropical bird species (32). This result agrees with other studies that refer to these four taxa as the “core microbiome” of wild avian species (33). Fusobacteria appeared at lower relative abundances but also occur in wild birds (34). This may reflect transient colonization, or the anaerobic microenvironments present within deeper oral tissues, although the oronasal cavity is generally more aerobic than the distal intestinal tract.

While the core microbiome is broadly conserved across avian microbiota compartments, the functional roles and metabolic activity of specific taxa may differ substantially between oral and intestinal environments (35, 36). The oro-nasal cavity represents a distinct ecological niche with unique pH, oxygen availability, and nutrient composition that likely shape community assembly and function differently than the gastrointestinal tract (37). Therefore, interpretations of metabolic pathways and bacterial functions derived from gut microbiota studies should be extrapolated to oral communities with caution. The bacterial composition we observed in oral samples includes taxa commonly recovered from avian microbiota. While some of these taxa are reported to possess metabolic capabilities related to plant material degradation (based primarily on studies of intestinal microbiota) (38), the functional significance of these taxa in the oral cavity remains unclear. The presence of diverse bacterial genera in oral swabs likely reflects environmental and dietary exposure, though direct inference about diet from oral microbiota composition requires caution, as oral bacterial communities differ substantially from intestinal communities (35, 36).

The presence of Actinomycetia (phylum Actinobacteria) is commonly associated with the degradation of complex carbohydrates and fiber, and suggests exposure to diverse environmental substrates, possibly including the organisms or plant material present in fish prey and scavenged carrion (38).

Among Firmicutes, Clostridiaceae and Peptostreptococcaceae are commonly detected in anaerobic environments and have been characterized primarily from intestinal microbiota studies. Their presence in oral samples may indicate anaerobic microenvironments in the oral cavity or transient colonization from ingested material. Lachnospiraceae and Oscillospiraceae, while known for fiber-degrading capacity in intestinal environments, have not been extensively studied in oral communities; their functional roles in the oral cavity remain uncharacterized. This community pattern is typical of herbivorous birds (e.g., grouse, geese, ratites), seed-eating birds, and omnivores that regularly ingest plant matter (39, 40).

The Negativicutes community, largely represented by Veillonellaceae, acts as secondary fermenters that metabolize lactate produced by other gut bacteria, thereby enhancing energy recovery from plant-based substrates such as grains, seeds, and fruits (41, 42). In addition, the presence of Porphyromonadaceae and Bacteroidaceae is consistent with diets that include both plant material and protein-rich resources, such as invertebrates or carrion, and is characteristic of birds with omnivorous or opportunistic feeding strategies (40). Notably, the detection of Actinomycetia, Clostridiaceae, and Bacilli, albeit at lower abundances, may also link to fish consumption (41).

The oral bacterial community reflects exposure to diverse environmental and dietary sources, though firm conclusions about diet require additional data from fecal or intestinal samples.

Alpha diversity analysis revealed high genus richness (285–363 genera per sample) together with comparably high Shannon values (H = 4.02–4.10), consistent with genus-level bacterial communities in which no single genus overwhelmingly dominates and in which a large proportion of the detected genera contribute measurably to overall diversity. This was corroborated by Simpson’s dominance index, which was low across all samples (*D* = 0.047–0.059, equivalent to an inverse Simpson index of ~17–21), confirming that no single genus achieved strong numerical dominance in any sample. Richness and Simpson’s dominance varied more between samples than Shannon diversity did, indicating that differences among samples were driven mainly by the number of genera detected and by the relative weight of the few most abundant genera (Veillonella, Megamonas and Corynebacterium were the three most abundant genera in all four samples), rather than by large differences in overall community evenness. Collectively, the results indicate that sample 1C harboured the comparatively most even bacterial community structure despite having the lowest richness; sample 5C, despite having the highest richness, showed the greatest dominance by a subset of genera (lowest evenness); and sample 6C showed the lowest overall genus-level diversity among the four samples, consistent with the particularly high relative abundance of Veillonella (18.8%) observed in this sample.

Beta diversity showed moderate compositional variation among samples (Bray-Curtis distances ranging from 0.151 to 0.292). The PCoA ordination did not show visible clustering associated with disease status or herpesvirus positivity. Sample 6C (herpesvirus-negative, sick) appeared compositionally more distinct from the other samples in both the PCoA and the Bray-Curtis distance matrix (Supplementary Table 3), but given the very limited sample size and the absence of formal statistical comparisons, this observation cannot be interpreted as evidence of a biological association with health or herpesvirus status.

Regarding the viral population revealed in this study, the bacteriophages (Caudoviricetes, Malgrandaviricetes, and Megaviricetes) were the most abundant taxa in all samples. These include DNA viruses that dominate the gut virome and infect a wide range of bacterial hosts, including those from the phyla Proteobacteria and Bacteroidetes, which we also detected in our samples (42, 43).

Using a metagenomic approach, we also identified sequences showing amino-acid similarity, based on BLASTx similarity searches, to members of the genus *Aviadenovirus* (family Adenoviridae) and to the family Hepadnaviridae.

However, robust phylogenetic trees could not be obtained, primarily due to the short length of the retrieved contigs and the fact that the conserved genomic regions conventionally used for phylogenetic classification of these virus families were not captured in the sequencing data. Complete reference genomes for *Adenoviridae* and *Hepadnaviridae* are available in public databases; the limiting factor was therefore not reference data availability, but the fragmentary nature of our contigs, which precluded alignment across these informative regions.

It is important to acknowledge the inherent limitations of similarity-based taxonomic assignment, particularly for short viral sequences, where the lack of robust phylogenetic placement can hinder confident taxonomic classification and may lead to misassignment or overinterpretation of sequence identity. It should be noted that BLAST-based similarity searches, while useful for preliminary identification, can provide only approximate taxonomic relationships for divergent sequences and do not necessarily reproduce true phylogenetic relationships (44).

Despite this limitation, we report the closest available BLAST-based classifications for both *Adenoviridae* and *Hepadnaviridae*, while emphasizing that these assignments remain tentative and are based on similarity to the closest matching reference sequences currently available.

Few studies have reported the presence of adenovirus- and hepadnavirus-related sequences in wild birds, and their role is largely unknown. In this study, we reported Adenovirus-related sequences in one healthy animal (1C) and one sick (9C). Adenovirus causes conditions ranging from subclinical to severe disease (45). However, since the presence of this virus was identified only after sample collection, typical clinical signs of adenovirus infection were not specifically investigated.

Aviadenovirus infections are frequently subclinical in healthy birds, with clinical disease typically emerging only following stress, immunosuppression, or concurrent infection (43–48); the genus is nonetheless an important cause of morbidity and mortality in poultry (49) and pigeons (50). Similarly, the genus Avihepadnavirus comprises viruses infecting various avian species, including ducks, geese, cranes, herons, storks, and parrots (51). Duck hepatitis B virus, the most studied strain, typically establishes asymptomatic, persistent infection in healthy adult ducks (52), although it can occasionally result in disease that is more overt. These observations support the interpretation that the adenovirus- and hepadnavirus-related sequences we detected, present in both a healthy and a clinically affected individual, do not, on their own, indicate an association with the clinical signs observed in this study. Since these studies (43–48, 52) report the detection in apparently healthy or asymptomatic birds, it may indicate that these viruses may circulate in avian populations in the absence of overt clinical disease.

Despite limitations such as the small sample size, and the absence of parallel sampling from cohabiting species, we confirmed the circulation of herpesvirus in frigatebirds from French Guiana. Although these limitations prevent a definitive interpretation, it is important to share the results of the metagenomic analysis, as they contribute valuable information on the bacterial and viral populations harbored by wild animals. Such data may also help improve our future understanding of the emergence of symptoms and the role of coinfecting viruses.

## 5 Conclusion

This study provides evidence of continued herpesvirus circulation in Magnificent Frigatebirds from French Guiana. We detected a DdDp sequence closely related to that reported previously in this population in sample 1C and a genetically divergent DdDp sequence in sample 9C. These findings do not establish whether either herpesvirus caused the observed clinical signs.

The detection of herpesviruses in apparently healthy individuals, together with their absence in blood, indicates that herpesvirus detection may be associated with the observed clinical condition, while the present data do not establish a causal contribution to the clinical signs or mortality observed. Metagenomic analysis revealed comparable microbial profiles in sick and healthy animals. The main limitations of the study were the small sample size, which reflects the difficulty of sampling these animals in their natural habitat, and the single-timepoint sampling design, constrained by logistical challenges. Despite these limitations, the study provides the first metagenomics-based characterization of frigatebird microbiota and highlights the need for integrated pathogen surveillance and environmental health monitoring. Expanded longitudinal sampling and multi-species comparative studies will be critical for understanding the factors associated with mortality and developing evidence-based conservation strategies for this vulnerable population.

## Data Availability

All sequences produced in this study were submitted to GenBank database. Accession numbers: PZ016842, PZ016843, PZ016846, PZ016847, PZ818782, PZ818783. Raw Reads were submitted to and SRA database (Sequence Read Archive): SRR40255407-SRR40255410, BioSample accessions (SAMN): SAMN62532914-SAMN62532917, and BioProject accession (PRJNA): PRJNA1515119.

## Supplementary material

SUPPLEMENTARY FIGURE 1 Heatmap illustrating the relative abundance (percentage) of contigs classified at the phylum level for each individual.

SUPPLEMENTARY FIGURE 2 Heatmap illustrating the relative abundance (percentage) of contigs classified at the class level for each individual.

SUPPLEMENTARY FIGURE 3 PCoA of the bacterial microbial community (Bray-Curtis).

SUPPLEMENTARY FIGURE 4 Phylogenetic Maximum Likelihood tree was built using an amino-acid alignment of complete UL5 protein from Alphaherpesvirus. The analysis included the sequence obtained from samples 1C. Branch support values were calculated using 1,000 bootstrap replicates and model chosen by ModelTest (LG+I+G).

SUPPLEMENTARY FIGURE 5 Phylogenetic Maximum Likelihood tree was built using an amino-acid alignment of complete UL6 protein from Alphaherpesvirus. The analysis included the sequence obtained from samples 1C. Branch support values were calculated using 1,000 bootstrap replicates and model chosen by ModelTest (LG+I+G).

SUPPLEMENTARY FIGURE 6 Phylogenetic Maximum Likelihood tree was built using an amino-acid alignment of the complete DNA-directed DNA polymerase (DdDp/UL30) protein sequence from Alphaherpesvirus. The analysis included the sequence obtained from sample 1C. Branch support values were calculated using 1,000 bootstrap replicates and model chosen by ModelTest (JTT+F+R5).

SUPPLEMENTARY TABLE 1 List of sequences used to build the phylogenetic tree using DdDp protein sequences of Alphaherpesviruses.

SUPPLEMENTARY TABLE 2 Alpha Diversity Indices of Bacterial Communities at Genus Level.

SUPPLEMENTARY TABLE 3 Beta Diversity: Pairwise Bray-Curtis Distances Among Bacterial Communities.
