# Supplementary material for: Metagenomic characterization of microbial communities associated with alphaherpesvirinae detection in magnificent frigatebirds from French Guiana
Source: Front Vet Sci. 2026 Oct 14;13:1866497. doi: 10.3389/fvets.2026.1866497 (PMC13714930; doi:10.3389/fvets.2026.1866497)
Supplement: Supplementary file 1 [file Table_1.DOCX]

**Supplementary Table 1. **List of sequences use to build the phylogenetic tree using DdDp protein sequences of Alphaherpesviruses****

| **Accesion numbers** | **Sequence name** |
| --- | --- |
| AAT79466.1 | DNA polymerase [Vulture herpesvirus] |
| YP_009342373.1 | DNA polymerase catalytic subunit [Spheniscid alphaherpesvirus 1] |
| YP_068333.1 | DNA polymerase catalytic subunit [Suid alphaherpesvirus 1] |
| YP_010795408.1 | polymerase catalytic subunit [Suid alphaherpesvirus 1] |
| YP_010795070.1 | DNA polymerase catalytic subunit [Equid alphaherpesvirus 8] |
| YP_002333511.1 | DNA polymerase catalytic subunit [Equid alphaherpesvirus 9] |
| YP_053075.1 | DNA polymerase catalytic subunit [Equid alphaherpesvirus 1] |
| NP_045247.1 | DNA polymerase catalytic subunit [Equid alphaherpesvirus 4] |
| YP_009054933.1 | DNA polymerase catalytic subunit [Equid alphaherpesvirus 3] |
| YP_010801429.1 | DNA polymerase catalytic subunit [Equid herpesvirus 6] |
| YP_010795345.1 | DNA polymerase [anatid alphaherpesvirus 1] |
| YP_003331549.1_ | DNA polymerase catalytic subunit [Felid alphaherpesvirus 1] |
| YP_009252254.1 | DNA polymerase catalytic subunit [Canid alphaherpesvirus 1] |
| YP_010794862.1 | DNA polymerase catalytic subunit-like protein [Phocid alphaherpesvirus 1] |
| NP_954917.1 | UL30 DNA polymerase, catalytic subunit [Bovine alphaherpesvirus 5] |
| YP_009664649.1 | DNA polymerase catalytic subunit [Bubaline alphaherpesvirus 1] |
| YP_010087572.1 | DNA polymerase catalytic subunit [Cervid alphaherpesvirus 3] |
| YP_010794995.1 | DNA polymerase catalytic subunit [Cervid alphaherpesvirus 1] |
| YP_010374217.1 | DNA polymerase [Bovine herpesvirus type 1.1] Alphaherpesvirinae |
| YP_010794925.1 | DNA polymerase catalytic subunit [Cervid alphaherpesvirus 2] |
| YP_010798662.1 | DNA polymerase catalytic subunit [Caprine alphaherpesvirus 1] |
| YP_010084960.1 | DNA polymerase catalytic subunit [Beluga whale alphaherpesvirus 1] |
| YP_009137182.1 | DNA polymerase catalytic subunit [Human alphaherpesvirus 2] |
| NP_042931.1 | DNA polymerase catalytic subunit [Human betaherpesvirus 6A] |
| YP_010802597.1_ | DNA polymerase catalytic subunit [Psittacid alphaherpesvirus 5] |
| YP_010795260.1 | DNA polymerase catalytic subunit [Gallid alphaherpesvirus 1] |
| YP_182359.1 | DNA polymerase catalytic subunit [Gallid alphaherpesvirus 1] |
| YP_010801579.1 | DNA polymerase catalytic subunit [Cacatuid alphaherpesvirus 2] Alphaherpesvirinae |
| NP_944403.1 | DNA polymerase catalytic subunit [Psittacid alphaherpesvirus 1] Alphaherpesvirinae |
| YP_009176907.1 | DNA polymerase catalytic subunit [Testudinid alphaherpesvirus 3] |
| YP_010795546.1 | F-UL30 DNA polymerase [Chelonid alphaherpesvirus 5] |
| YP_009207091.1_ | DNA polymerase catalytic subunit [Hawaiian green turtle herpesvirus] |
| NP_077443.1 | DNA polymerase catalytic subunit [Cercopithecine alphaherpesvirus 9] |
| NP_040151.1 | DNA polymerase catalytic subunit [Human alphaherpesvirus 3] |
| NP_073324.1 | DNA polymerase catalytic subunit [Meleagrid alphaherpesvirus 1] |
| YP_001033959.1 | DNA polymerase catalytic subunit [Gallid alphaherpesvirus 2] |
| YP_010795626.1 | DNA polymerase [Gallid alphaherpesvirus 3] |
| NP_066862.1 | DNA polymerase catalytic subunit [Gallid alphaherpesvirus 3] |
| YP_009352938.1 | DNA polymerase catalytic subunit [Columbid alphaherpesvirus 1] |
| YP_009046528.1 | DNA polymerase catalytic subunit [Falconid herpesvirus 1] |
| YP_010798834.1 | DNA polymerase [Macropodid alphaherpesvirus 2] |
| YP_009227263.1 | DNA polymerase [Macropodid alphaherpesvirus 1] |
| YP_010798748.1 | DNA polymerase catalytic subunit [Bovine alphaherpesvirus 2] |
| YP_009230161.1 | DNA polymerase [Leporid alphaherpesvirus 4] |
| YP_003933809.1 | DNA polymerase catalytic subunit [Saimiriine alphaherpesvirus 1] |
| YP_009361907.1 | DNA polymerase catalytic subunit [Ateline alphaherpesvirus 1] |
| YP_010801511.1 | DNA polymerase catalytic subunit [pteropodid alphaherpesvirus 2] |
| YP_009042092.1 | DNA polymerase catalytic subunit [Pteropodid alphaherpesvirus 1] |
| NP_851890.1 | DNA polymerase catalytic subunit [Macacine alphaherpesvirus 1] |
| YP_010797404.1 | DNA polymerase catalytic subunit [Macacine alphaherpesvirus 1] |
| YP_010797330.1 | DNA polymerase catalytic subunit [Macacine alphaherpesvirus 1] |
| YP_164473.1 | DNA polymerase catalytic subunit [Cercopithecine alphaherpesvirus 2] |
| YP_443877.1 | DNA polymerase catalytic subunit [Papiine alphaherpesvirus 2] |
| YP_009137105.1 | DNA polymerase catalytic subunit [Human alphaherpesvirus 1] |
| YP_009011017.1 | DNA polymerase catalytic subunit [Chimpanzee herpesvirus strain 105640] |

**Supplementary Table 2. Alpha Diversity Indices of Bacterial Communities at Genus Level**

| **Sample** | **Shannon** | **Simpson** | **Richness** |
| --- | --- | --- | --- |
| **1C** | 4.101882 | 0.0472 | 285 |
| **5C** | 4.085696 | 0.0590 | 363 |
| **6C** | 4.015474 | 0.0586 | 324 |
| **9C** | 4.087190 | 0.0544 | 314 |

**Supplementary Table 3. Beta Diversity: Pairwise Bray-Curtis Distances Among Bacterial Communities**

|  | **1C** | **5C** | **6C** | **9C** |
| --- | --- | --- | --- | --- |
| **1C** | 0.0 | 0.209 | 0.284 | 0.151 |
| **5C** | 0.209 | 0.0 | 0.292 | 0.215 |
| **6C** | 0.284 | 0.292 | 0.0 | 0.265 |
| **9C** | 0.151 | 0.215 | 0.265 | 0.0 |
